# Serum Zinc Level in Asthmatic and Non-Asthmatic School Children

**DOI:** 10.3390/children5030042

**Published:** 2018-03-16

**Authors:** Atqah AbdulWahab, Aseel Zeidan, Tony Avades, Prem Chandra, Ashraf Soliman

**Affiliations:** 1 Department of Pediatrics, Hamad Medical Corporation, P.O. Box 3050, Doha, Qatar; Aseel.Zeidan@gmail.com (A.Z.); atsoliman@yahoo.com (A.S.); 2 Weill Cornell Medicine, P.O. Box 3050, Doha, Qatar; 3 Department Laboratory Medicine and Pathology, Hamad Medical Corporation, P.O. Box 3050, Doha, Qatar; tonyavades@gmail.com; 4 Medical Research Center, Hamad Medical Corporation, P.O. Box 3050, Doha, Qatar; Pchandra@hamad.qa

**Keywords:** asthma, school asthmatic children, serum zinc

## Abstract

Asthma is one of the most common chronic disorders among children. Zinc (Zn) is an essential dietary antioxidant and may have a special role in assisting the airways of asthmatic subjects. The primary objective of this study was to measure serum Zn levels among asthmatic school children and to compare this to the serum Zn level in non-asthmatic children. The secondary objective was to investigate the relationship between Zn levels and the degree of asthma control. A cross-sectional study following forty asthmatic children and forty matched non-asthmatic children of both genders was conducted. Weight, height, body mass index (BMI), BMI Z-scores, serum Zn, hemoglobin, total protein, and albumin concentrations were measured in both groups. Serum immunoglobulin E (IgE) levels, the forced expiratory volume in 1 second (FEV1), and dosage of inhaled steroids were measured in asthmatic school children. The results show the mean Zn level among asthmatic children was 12.78 ± 1.8 μmol/L. Hypozincemia was detected in four asthmatic children. Asthma and control groups were matched in age, gender, and BMI Z score (*p* > 0.05). No significant difference was observed in Zn levels, hemoglobin, albumin, and total protein between both groups (*p* > 0.05). Among asthmatics, Zn levels were not significantly associated with the degree of asthma control (well controlled, mean Zn = 12.9 ± 1.5, partially controlled, mean Zn = 11.9 ± 1.6, and uncontrolled, mean Zn = 3.62 ± 2.2) (*p* = 0.053). The Zn level was not correlated with the FEV1 Z score. There was no significant association between Zn level and the dosage of inhaled steroids or IgE concentrations (*p* > 0.05). The findings show that Zn may not play a major role in the degree of asthma control. Larger studies are needed to confirm these results.

## 1. Introduction

Asthma is the most common chronic disease in childhood, and the prevalence has increased considerably in recent decades [1,2]. According to international guidelines, the ultimate goal of asthma management is to control the disease in terms of symptoms, pulmonary function, preventing asthma exacerbations and avoiding adverse effects from asthma medications. The main controller medications are inhaled corticosteroids that switch off the inflammation of asthmatic airways [3,4].

Zinc (Zn) is a trace mineral involved in many functions in the body [5]. Zn, an essential dietary metal, plays special roles in the conducting airways and contributes partly to the structure and function of many biological enzymes. It also regulates ion transporters relevant to pulmonary diseases [6,7,8,9]. Zn is known to exhibit powerful antioxidant activity in the lungs and several body organs [6,7,8,9]. Reactive oxygen species (ROS) have a role in initiating inflammation in asthmatic airways. Excessive ROS production in asthma leads to an oxidant-antioxidant imbalance in airways [10]. It is possible that Zn deficiency can disturb the equilibrium between type 1 and type 2 T helpers, [11] which leads to increased inflammation and eosinophilia. This is the same mechanism detected in allergic airway hypersensitivity [12].

Chronic asthmatics take prophylactic inhaled steroids daily, which may have an important contributing effect on serum Zn status. Such a study has not been reported in the literature, but there are some studies that demonstrated a relationship between oral steroids and Zn levels in normal humans, asthmatic patients, burn patients, and patients with rheumatoid arthritis [13,14,15].

The objective of our study was to measure the serum Zn level among asthmatic school children aged 7–14 years and to compare it to the serum Zn level in non-asthmatic children. The secondary objective was to investigate the association, if any, of Zn levels with the degree of asthma control and dosage of inhaled steroids.

## 2. Materials and Methods

### 2.1. Study Design and Population

A cross-sectional study was conducted on 40 asthmatic school children attending the pediatric pulmonary clinics and 40 non-asthmatic school children from general pediatric clinics at Hamad Medical Corporation (HMC), Doha, Qatar, between January 2015 and May 2015. The following inclusion criteria for selecting asthmatic school children were used: (i) aged between 7 and 14 years and able to perform a spirometry and (ii) the diagnosis of asthma was based on a physician’s diagnosis according to the American Thoracic Society (ATS) guidelines in accordance with guidelines of the Global Initiative for asthma and the International Study of Asthma and Allergies in Childhood(ISAAC) questionnaire [16,17]. Asthma control was assessed using the Asthma Control Test (ACT) that includes five items (activity limitations, shortness of breath, nocturnal symptoms, rescue medication, and overall control in the past 4 weeks). Each item was scored from 1 (worst) to 5 (best). The ACT ranged from 5 to 25 (better indicated by higher values). A score of >20 indicates well-controlled asthma, 16–19 partially controlled, and <16 uncontrolled asthma [18]. Healthy controls with matched age and gender (who dif not have recent or previous respiratory symptoms) were selected from the General pediatric clinic of the same hospital. Neither lung function or total IgE (immunoglobulin E) levels were measured for control subjects. We only measured the serum Zn level, hemoglobin, total protein and albumin concentrations for control subjects and then compared with the levels with those of asthma subjects. None of the subjects in either the asthmatic or control group had an acute respiratory infection for more than 4 weeks prior to enrollment. In addition, none of the asthmatic children had acute asthma exacerbation prior to enrollment. All subjects with significant advanced liver disease, diabetes mellitus those requiring insulin therapy, renal failure, malignancies, or collagen vascular disease or medications (both oral steroids and nonsteroidal anti-inflammatory drugs) were excluded from the study. In addition, we excluded all vegetarian subjects. This study was reviewed and approved by the local ethics committee at the Medical Research Center at HMC (Joint Institutional Review Board (JIRB), reviewed and approved on 23 October 2014; IRB Number: 14-00136). Written informed consent was obtained from the parents or guardians of the children and assent was obtained from children 9 years old and older.

### 2.2. Anthropometric Measurements

Weight measurements were recorded using a digital electronic platform scale, and a standing height measurement without shoes was taken using a stadiometer. Body mass index (BMI) was calculated by dividing weight in kg by height squared in meters adjusted for age and gender. The standard deviation [Z] score for BMI was calculated.

Patients’ spirometry tests were performed in the respiratory laboratory unit in accordance with the standards of the American Thoracic Society/European Respiratory Society task force: standardization of lung function testing [19]. Spirometry was performed in patients old enough to perform this adequately. The highest of three appropriate measurements was recorded. Forced vital capacity (FVC; in liters and % predicted), forced expiratory volume in 1 s (FEV1; in liters and % predicted), and FEV1/FVC were measured using a flow-sensing spirometer (Sensor Medicus Model V6200, Hochberg, Germany.

### 2.3. Biochemical Analysis

Whole blood (5 mL) in a trace metal plain tube was collected from subjects (non-fasting blood sample) for measuring albumin, protein, hemoglobin and Zn. These measurements were determined by an atomic absorption spectrophotometer. An experienced senior laboratory technician performed the tests. Total IgE levels were measured in the school children with asthma.

### 2.4. Sample Size

An adequate sample was determined based on the primary outcome variables of the serum Zn level between asthmatic and healthy control groups. As per a literature review [20,21], the mean Zn levels in asthmatic and healthy control groups were found to be 10.8 ± 2.5 μmol/L and 12.5 ± 1.8 μmol/L, respectively. With 90% power and at two-sided 0.05 level of significance, the required sample size was 35 patients in each group. To account for the proportion of dropouts and non-participation, 40 patients altogether were needed for enrollment.

### 2.5. Statistics

Categorical data are expressed as a frequency along with percentage and continuous data values presented as mean ± SD (standard deviation). Descriptive statistics summarized the demographic, BMI Z-score, serum zinc, spirometry measures and other characteristics of the participants. Association between two or more qualitative variables was analyzed using Chi-square or Fisher exact tests as appropriate. Unpaired *t*-tests compared the mean of quantitative variables between the two independent groups. The quantitative data mean across different degrees of asthma control (uncontrolled, partially controlled and controlled) was compared using one-way analysis of variance (ANOVA) and Kruskal Wallis tests as appropriate depending on the results of the normality test. Linear regression and Pearson’s correlation coefficient were applied to estimate and assess the prediction and strength of the relationship between the Zn level and FEV1. A *p*-value of less than 0.05 was considered to be statistically significant. All statistical analyses were done using the statistical package SPSS 22 (SPSS Inc., Chicago, IL, USA).

## 3. Results

**Figure 1:**
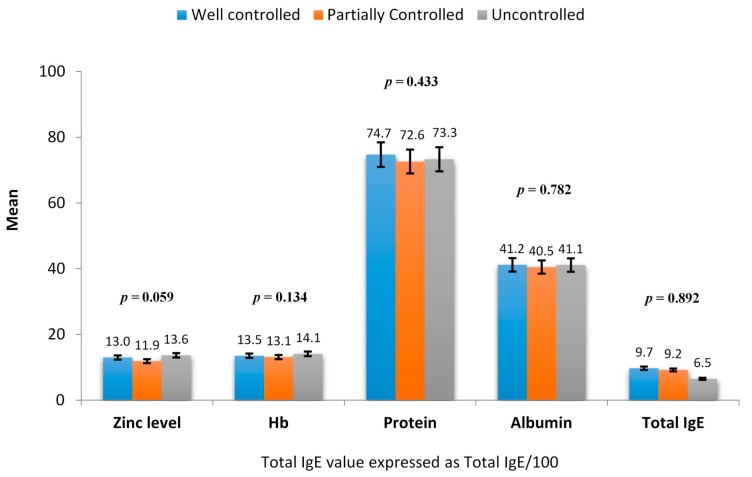
Laboratory characteristics among asthmatic children in relation to asthma control. IgE: Immunoglobulin E; Hb: hemoglobin, *p*—*p-*value.

Eighty children including 40 asthmatic school children (aged 10.92 ± 1.81 years) and 40 matched non-asthmatic school children (aged 10.89 ± 1.87 years) were studied. Demographics, anthropometric characteristics and laboratory characteristics of asthmatic and healthy children are shown in Table 1. Asthmatic and non-asthmatic groups were matched for age, gender, and BMI Z score (*p* > 0.05). No significant difference was observed in Zn levels, albumin and total protein between both groups (*p* > 0.05). Mean hemoglobin levels were noted to be significantly higher in the asthmatic group compared to the non-asthmatic group (*p* = 0.040). Our reference value for normal Zn levels was between 10.1–16.83 μmol/L according to the standard laboratory reference in our institution. The mean Zn level among asthmatic children was 12.78 ± 1.8 μmol/L, and four patients showed Zn deficiency. The mean serum Zn level among non-asthmatic children was 13.0 ± 1.52 μmol/L, one of which had a Zn deficiency. Among asthmatic children, Zn levels were not significantly associated with the degree of asthma control (well controlled, *n* = 17 patients; partially controlled, *n* = 15 patients and uncontrolled, *n* = 8 patients) (*p* > 0.05) (Figure 1). All 40 asthmatic children performed spirometry tests, and the mean values observed for these parameters were FEV1% 84.9 ± 14.2, FVC% 92 ± 12, and FEV1/FVC 91.1 ± 8.7, respectively, and no significant correlation was found between the Zn level and FEV1% (Pearson correlation *r* = 0.220, *p* = 0.172), FVC% (*r* = 0.210, *p* = 0.194), and FEV1/FVC (*r* = 0.135, *p* = 0.405). All of the asthmatic children were taking inhaled steroids. The mean dosage of inhaled steroids did not differ among the different groups showing varying severity. The well-controlled asthma group had a mean dosage of 258.8 ± 134.9 μg/day, the partially controlled asthma group had a mean dosage of 310 ± 154.9 μg/day, and the uncontrolled group had a mean dosage of 243 ± 105.1 μg/day (*p* = 0.415). The mean total IgE value of the asthma group was 890.54 ± 1565.9 ku/L. There was no significant association observed between total IgE and Zn levels (*r* = − 0.082; *p* = 0.614).

## 4. Discussion

In the present study, we evaluated the Zn status in asthmatic school children. There was no difference in the mean serum Zn levels between asthmatic and non-asthmatic school children.

Zn is considered a gatekeeper of immune function and is important in immunological reactions such as the inflammatory response and the oxidative stress response [22].

Hypozincemia was detected in four asthmatic children and in one child in the control group (statistically not significant). Zn deficiency has been suggested to play a role in the pathogenesis, control and severity of asthma because of its antioxidant, anti-apoptotic and anti-inflammatory effects in the respiratory epithelium. With respect to T cells, a disturbed ratio of Th1 and Th2 cells in favor of Th2-driven allergic reactions is a well-known consequence of Zn deficiency [23]. Moreover, the pro-tolerogenic immunoreaction is triggered by long-lasting changes in intracellular Zn levels due to the induction of regulatory T cells and the dampening of pro-inflammatory Th17 and Th9 cells [24].

Several studies reported conflicting relationship between Zn status and asthma. Similar to our finding, Kocyigit et al., reported plasma Zn, copper and albumin did not significantly differ in asthmatic children when compared to controls (*p* > 0.05) [25]. Other investigators found no significant decrease in Zn level in adult asthmatic patients [26]. In addition, other investigators found no association between erythrocyte Zn levels and severity, duration of follow-up, and control of the asthma [27]. In contrast to our findings, a number of investigators have reported that the serum Zn level tended to be lower in patients with bronchial asthma [13,20,21,28,29]. In a study from Iran where Zn deficiency seems to be a common condition (a prevalence of 13.7% in healthy children), Zn deficiency appeared to have a significant role in asthma severity [28]. Ghaffari J. et al. confirmed that administering Zn to asthmatic children with a low initial serum Zn produced significant improvements in clinical asthma symptoms and lung function [30].

Various environmental factors, including individual characteristics such as age, gender and genetic predisposition, lifestyle-related factors, and nutrient-related factors such as dosage, route, duration of the exposure and the presence and variety of elements, affect the epigenome and are termed the “interactome”. However, the interactome is difficult to estimate since drinking water and soil might contain different amounts of each trace element depending on the region [31].

In the present study, we did not find an association between Zn levels and the degree of asthma control. This is in agreement with a previous study by Picado et al., who reported no association between either dietary intake or plasma/serum levels of micronutrients/antioxidants and asthma severity [32].

All of the asthmatic school children were taking inhaled steroids. We found no significant association between Zn levels and the dose of inhaled steroids. A similar finding was reported by Goldey et al., who found no correlation between the serum Zn level and the use of steroids in asthmatic children [33].

A study from Turkey reported low serum selenium, Zn and magnesium during acute asthma exacerbation, which increased after inhaled steroid treatment [34]. In another study Raeve et al. showed that corticosteroids decreased oxidant levels by decreasing the number of oxidant-generating cells present in the asthmatic airway mucosa and reducing macrophage oxidant production [35]. In contrast to these findings, several studies showed that the mean serum Zn level was lower in children using steroids compared to non-users [27,36,37,38]. They suggested that steroids may increase urinary excretion of Zn-reducing serum, which would reduce the serum Zn level.

Although it has been found that hypo-zincemic allergic asthmatic patients had a higher mean total IgE, we did not find an association between Zn levels and IgE levels among our asthmatic children [36].

The limitations in the present study are the low number of asthmatic school children, especially those with low Zn levels, resulting in a lack of generalizability of our findings to other (non Arab) populations, and the use of serum Zn is less accurate than evaluating the tissue Zn level. In addition, the present study did not assess confounding factors such as environmental tobacco that may affect the Zn levels or the association between Zn and lung function or asthma control.

## 5. Conclusions

Serum Zn may not play a major role in asthma control. However, further multicenter studies with greater sample sizes and adequate statistical power are required to prove the findings of the present study.

## Figures and Tables

**Table 1:** Demographics and laboratory results among cases and controls.

Variable		Asthma	Non-Asthma	*p*-Value *
Age (years)		10.92 ± 1.807	10.89 ± 1.873	0.934
Gender	Male	34 (85%)	32 (80%)	0.556
	Female	6 (15%)	8 (20%)	
BMI Z scores		1.12 ± 1.35	0.92 ± 1.35	0.500
Hemoglobin (g/dL)		13.46 ± 1.07	12.98 ± 0.92	0.040 *
Protein (g/L)		73.60 ± 4.48	73.23 ± 6.75	0.770
Albumin (g/L)		40.95 ± 2.87	42.93 ± 7.05	0.105
Zinc (μmol/L)		12.69 ± 1.80	13.0 ± 1.52	0.388

BMI—Body mass index. Quantitative and qualitative values presented as the mean ± SD (standard deviation) and frequency along with %. * Unpaired *t*-test and Chi-square *t*-test were used to compute the respective *p*-values.

## References

[1] (2011). Centers for Disease Control and Prevention: National Center for Health Statistics, National Health Interview Survey Raw Data.

[2] Gelfand E.W. (2009). Pediatric asthma: A different disease. Proc. Am. Thorac. Soc..

[3] Reddel H.K., Bateman E.D., Becker A., Boulet L.P., Cruz A.A., Drazen J.M., Haahtela T., Hurd S.S., Inoue H., De Jongste J.C. (2015). A summary of the new GINA strategy: A roadmap to asthma control. Eur. Respir. J..

[4] Castro-Rodriguez J.A., Rodrigo G.J., Rodriguez-Martinez C.E. (2015). Principal findings of systematic reviews for chronic treatment in childhood asthma. J. Asthma.

[5] Prasad A.S. (1996). Zinc deficiency in women, infants, and children. J. Am. Coll. Nutr..

[6] Zalewski P.D. (2006). Zinc metabolism in the airway: Basic mechanisms and drug targets. Curr. Opin. Pharmacol..

[7] Childers M., Eckel G., Himmel A., Caldwell J. (2007). A new model of cystic fibrosis pathology: Lack of transport of glutathione and its thiocyanate conjugates. Med. Hypotheses.

[8] Zsembery A., Fortenberry J.A., Liang L., Bebok Z., Tucker T.A., Boyce A.T., Braunstein G.M., Welty E., Bell P.D., Sorscher E.J. (2004). Extracellular zinc and ATP restore chloride secretion across cystic fibrosis airway epithelia by triggering calcium entry. J. Biol. Chem..

[9] Taylor C.G., Bray T.M. (1991). Effect of hyperoxia on oxygen free radical defense enzymes in the lung of zinc-deficient rats. J. Nutr..

[10] Nadeem A., Masood A., Siddiqui N. (2008). Oxidant-antioxidant imbalance in asthma: Scientific evidence, epidemiological data and possible therapeutic options. Ther. Adv. Respir. Dis..

[11] Hönscheid A., Rink L., Haase H. (2009). T-lymphocytes: A target for stimulatory and inhibitory effects of zinc ions. Endocr. Metab. Immune Disord. Drug Targets.

[12] Truong-Tran A.Q., Ruffin R.E., Foster P.S., Koskinen A.M., Coyle P., Philcox J.C., Rofe A.M., Zalewski P.D. (2002). Altered zinc homeostasis and caspase-3 activity in murine allergic airway inflammation. Am. J. Respir. Cell Mol. Biol..

[13] El-Kholy M.S., Gas Allah M.A., el-Shimi S., el-Baz F., el-Tayeb H., Abdel-Hamid M.S. (1990). Zinc and copper status in children with bronchial asthma and atopic dermatitis. J. Egypt Public Health Assoc..

[14] Kadrabová J., Mad’aric A., Podivínsky F., Gazdík F., Ginter F. (1996). Plasma zinc, copper and copper/zinc ratio in intrinsic asthma. J. Trace. Elem. Med. Biol..

[15] Peretz A., Neve J., Famaey J.P. (1989). Effects of chronic and acute corticosteroid therapy on zinc and copper status in rheumatoid arthritis patients. J. Trace Elem. Electrolytes Health Dis..

[16] American Thoracic Society (2004). Guidelines for assessing and managing asthma risk at work, school, and recreation. Am. J. Respir. Crit. Care Med..

[17] Weiland S.K., Björkstén B., Brunekreef B., Cookson W.O., von Mutius E., Strachan D.P., International Study of Asthma and Allergies in Childhood Phase II Study Group (2004). Phase II of the international study of asthma and allergies in childhood (ISAAC II): Rationale and methods. Eur. Respir. J..

[18] Nathan R.A., Sorkness C.A., Kosinski M., Schatz M., Li J.T., Marcus P., Murray J.J., Pendergraft T.B. (2004). Development of the asthma control test: A survey for assessing asthma control. J. Allergy Clin. Immunol..

[19] Miller M.R., Hankinson J., Brusasco V., Burgos F., Casaburi R., Coates A., Crapo R., Enright P., van der Grinten C.P., Gustafsson P. (2005). Standardization of spirometry. Eur. Respir. J..

[20] Kakarash T.A., Al-Rabat A. (2012). Zinc Status in Children with Bronchial Asthma. IPMJ.

[21] Vural H., Uzun K., Uz E., Koçyigit A., Cigli A., Akyol O. (2000). Concentrations of copper, zinc and various elements in serum of patients with bronchial asthma. J. Trace Elem. Med. Biol..

[22] Wessels I., Maywald M., Rink L. (2017). Zinc as a gatekeeper of immune function. Nutrients.

[23] Sprietsma J.E. (1997). Zinc-controlled Th1/Th2 switch significantly determines development of diseases. Med. Hypotheses.

[24] Kitabayashi C., Fukada T., Kanamoto M., Ohashi W., Hojyo S., Atsumi T., Ueda N., Azuma I., Hirota H., Murakami M. (2010). Zinc suppresses Th17 development via inhibition of STAT3 activation. Int. Immunol..

[25] Kocyigit A., Armutcu F., Gurel A., Ermis B. (2004). Alterations in plasma essential trace elements selenium, manganese, zinc, copper, and iron concentrations and the possible role of these elements on oxidative status in patients with childhood asthma. Biol. Trace Elem. Res..

[26] Urushidate S., Matsuzaka M., Okubo N., Iwasaki H., Hasebe T., Tsuya R., Iwane K., Inoue R., Yamai K., Danjo K. (2010). Association between concentration of trace elements in serum and bronchial asthma among Japanese general population. J. Trace Elem. Med. Biol..

[27] Arik Yilmaz E., Ozmen S., Bostanci I., Misirlioglu E.D., Ertan U. (2011). Erythrocyte zinc levels in children with bronchial asthma. Pediatr. Pulmonol..

[28] Khanbabaee G., Omidian A., Imanzadeh F., Adibeshgh F., Ashayeripanah M., Rezaei N. (2014). Serum level of zinc in asthmatic patients: A case-control study. Allergol. Immunopathol..

[29] Perrone L. (1987). Zinc and copper status of allergic children. Acta Paediatr. Scand..

[30] Ghaffari J., Khalilian A., Salehifar E., Khorasani E., Rezaii M.S. (2014). Effect of zinc supplementation in children with asthma: A randomized, placebo-controlled trial in Northern Islamic Republic of Iran. East. Mediterr. Health J..

[31] Samuelsson U., Oikarinen S., Hyoty H., Ludvigsson J. (2011). Low zinc in drinking water is associated with the risk of type 1 diabetes in children. Pediatr. Diabetes.

[32] Picado C., Deulofeu R., Lleonart R., Agustí M., Mullol J., Quintó L., Torra M. (2001). Dietary micronutrients/antioxidants and their relationship with bronchial asthma severity. Allergy.

[33] Goldey D.H., Mansmann H.C., Rasmussen A.I. (1984). Zinc status of asthmatic, prednisone-treated asthmatic, and non-asthmatic children. J. Am. Diet. Assoc..

[34] Uzuner N., Karaman Ö., Įoker C., Turgut F., Uzuner H., Önvural B. (2001). Serum trace element levels in bronchial asthma. Turk. Respir. J..

[35] De Raeve H.R., Thunnissen F.B., Kaneko F.T., Guo F.H., Lewis M., Kavuru M.S., Secic M., Thomassen M.J., Erzurum S.C. (1997). Decreased CuZn-SOD activity in asthmatic airway epithelium: Correction by inhaled corticosteroid in vivo. Am. J. Physiol..

[36] Agin K.h. (2015). A Survey on zinc status among chronic allergic asthma and in atopic phenotype. Int. J. Med. Toxic. Forensic Med..

[37] Flynn A., Pories W.J., Strain W.H., Hill O.A., Fratianne R.B. (1971). Rapid serum-zinc depletion associated with corticosteroid therapy. Lancet.

[38] Ellul-Micallef R., Galdes A., Fenech F.F. (1976). Serum zinc levels in corticosteroid-treated asthmatic patients. Postgrad. Med. J..

